# Growth and transcriptional response of wheat and rice to the tertiary amine BMVE

**DOI:** 10.3389/fpls.2023.1273620

**Published:** 2024-01-10

**Authors:** Jaspinder Singh Dharni, Yu Shi, Chi Zhang, Chris Petersen, Harkamal Walia, Paul Staswick

**Affiliations:** ^1^ Department of Agronomy and Horticulture, University of Nebraska, Lincoln, NE, United States; ^2^ School of Biological Sciences, University of Nebraska, Lincoln, NE, United States; ^3^ Kamterter Products, LLC, Waverly, NE, United States

**Keywords:** tertiary amine, germination, abiotic stress, transcriptome, growth, seed vigor, seed treatment

## Abstract

**Introduction:**

Seed vigor is largely a product of sound seed development, maturation processes, genetics, and storage conditions. It is a crucial factor impacting plant growth and crop yield and is negatively affected by unfavorable environmental conditions, which can include drought and heat as well as cold wet conditions. The latter leads to slow germination and increased seedling susceptibility to pathogens. Prior research has shown that a class of plant growth regulators called substituted tertiary amines (STAs) can enhance seed germination, seedling growth, and crop productivity. However, inconsistent benefits have limited STA adoption on a commercial scale

**Methods:**

We developed a novel seed treatment protocol to evaluate the efficacy of 2-(N-methyl benzyl aminoethyl)-3-methyl butanoate (BMVE), which has shown promise as a crop seed treatment in field trials. Transcriptomic analysis of rice seedlings 24 h after BMVE treatment was done to identify the molecular basis for the improved seedling growth. The impact of BMVE on seed development was also evaluated by spraying rice panicles shortly after flower fertilization and subsequently monitoring the impact on seed traits.

**Results:**

BMVE treatment of seeds 24 h after imbibition consistently improved wheat and rice seedling shoot and root growth in lab conditions. Treated wheat seedlings grown to maturity in a greenhouse also resulted in higher biomass than controls, though only under drought conditions. Treated seedlings had increased levels of transcripts involved in reactive oxygen species scavenging and auxin and gibberellic acid signaling. Conversely, several genes associated with increased reactive oxygen species/ROS load, abiotic stress responses, and germination hindering processes were reduced. BMVE spray increased both fresh and mature seed weights relative to the control for plants exposed to 96 h of heat stress. BMVE treatment during seed development also benefited germination and seedling growth in the next generation, under both ambient and heat stress conditions.

**Discussion:**

The optimized experimental conditions we developed provide convincing evidence that BMVE does indeed have efficacy in plant growth enhancement. The results advance our understanding of how STAs work at the molecular level and provide insights for their practical application to improve crop growth.

## Introduction

A critical factor determining plant growth and productivity is seed vigor, a complex trait encompassing seed dormancy, viability, germination rate, and seedling establishment. Seeds with low vigor are more susceptible to stress during germination, especially in unfavorable environments (Bewley et al., 2013; Finch-Savage and Bassel, 2016). Rapid and synchronous germination minimizes these detriments and maximizes uniformity at harvest time. Causes of low seed vigor include adverse environmental conditions during seed development and poor seed storage conditions after harvest (Bewley and Black, 2013; Zinsmeister et al., 2020). Seed vigor is an important trait that many plant breeding programs have aimed to improve through incorporation of increased genetic diversity (Lara-Fioreze et al., 2013; Dang et al., 2014; Luo et al., 2021).

A useful technology to reduce germination time and improve germination synchrony is seed priming. This most often involves controlled hydration of seeds to initiate early stages of germination, followed by drying treatments to suspend germination until planting (Osborne, 1983; Halmer, 2004; Hussain et al., 2016). A variety of stress, chemical and bioactive treatments of seeds have also been investigated to enhance seed germination, with or without typical seed priming protocols (Ashraf and Foolad, 2005; Ilyas, 2006; Singh, 2018; Rhaman et al., 2021). One mechanism behind the efficacy of seed priming may involve the enhanced activation of antioxidant defense systems, which help counter the toxic reactive oxygen species that accrue during germination, especially under stress conditions (Gill and Tuteja, 2010; Xu et al., 2011; Khaliq et al., 2015; Hussain et al., 2016; Zheng et al., 2016; Guo et al., 2022). On the other hand, primed seeds are more susceptible to deterioration before planting, reducing their storage longevity (Heydecker and Gibbins, 1977; Tarquis and Bradford, 1992; Chiu et al., 2002).

Several reports have suggested that a class of plant growth regulators known collectively as substituted tertiary amines (STAs) can enhance seedling development and increase crop productivity when used to treat seeds or growing plants (Jung et al., 1975; Hayman and Yokoyama, 1990; Keithly et al., 1990a, b; Keithly and Yokoyama, 1992; Ashraf and Foolad, 2005; Van Pelt, 2007; Qi et al., 2013; Banerjee et al., 2014; Yan, 2015; Wang et al., 2016; Gao et al., 2022). Low-dose application of these compounds to seeds prior to germination reportedly increases root growth and seedling establishment, while foliar application at later stages can enhance photosynthetic efficiency along with yield traits (US9464283B2, Kamtec LLC) (Yokoyama et al., 2015). A recent study reported that foliar application of DCPTA [2-(3,4-dichlorophenoxy) triethylamine] enhanced expression of genes associated with photosynthetic output (Gao et al., 2022).

Despite the positive examples of STAs, other field and greenhouse studies showed no or inconsistent benefits (Hartz et al., 1995; Davies et al., 2004; Singh, 2018). Thus, the early promise of STAs has generally not translated to commercial use. The reasons for the discrepancies in reported efficacy are not clear, although they could be related to differential plant response under variable environmental conditions, or variation in cultivar/genotype response to tertiary amines.

A significant deficiency in our current knowledge of STAs is an understanding of their action at the molecular level. While some effects of STAs are reminiscent of plant hormone activities, STA mode of action does not appear to simply mimic phytohormones (Gausman, 1991; Van Pelt, 2007). Understanding how STAs act at the molecular level could help to explain why positive results have been sporadic and might allow for their use in a more consistently beneficial way.

In this report we optimized conditions under which seed treatment with 2-(N-methyl benzyl aminoethyl)-3-methyl butanoate (BMVE) consistently enhanced rice (*Oryza sativa* L. ssp. Japonica, var. Kitaake) and wheat (*Triticum aestivum*, var. Ruth) seedling growth in the laboratory. We determined the physiological modifications triggered by BMVE seed treatment and examined whether the positive seedling effects were sustained through later vegetative growth under normal and drought conditions. We also investigated the transcriptional changes occurring in seedlings shortly after their treatment with BMVE to gain insight into the underlying molecular mechanism of BMVE actions. Finally, we explored whether BMVE treatment of seeds while they were developing on the plant enhanced germination and plant growth in the next generation. Results from this study show that BMVE has efficacy in improving plant growth when applied at the early seedling stage, and when sprayed on rice panicles during seed development.

## Materials and methods

### Seed treatment method

The formal name for BMVE is 2-(N-methyl benzyl aminoethyl)-3-methyl butanoate. Before each experiment, freshly prepared BMVE (100mM in ethanol) was used as a stock to further prepare the working solution in distilled water at the specified concentrations. Control solutions for seed treatments used the same volume of 100% ethanol that was used for the 100mM BMVE treatment. Before the treatment, seeds were imbibed for 24 hours in dark. At this point, uniform seeds having an emerged radical of about 2 mm were selected and treatment was applied by soaking the seeds in BMVE or control solution for 3 hours in dark. Following the treatment, seeds were kept on moist paper under dark conditions for an additional 24 hours. The temperatures used for the dark pre- and post-treatment periods was 23°C and 28°C for wheat and rice, respectively. Varieties used in this study were Kitaake (*Oryza sativa* L.), a Japonica rice commonly used in research worldwide and Ruth, a wheat (*Triticum aestivum*) variety developed for the great plains area of the United Sates.

### Measurements during early seedling growth

Following seed treatment, seeds were set to grow on wet tissue paper (six layers) in small plastic boxes. Boxes were covered with aluminum foil to maintain dark conditions until measurements. All the material including tissue paper, boxes, and water was sterilized before use. During the preliminary experiment that aimed at tracking wheat shoot growth until 9 days after growth, treated wheat seeds were grown in plastic trays containing small pots filled with autoclaved soil (greenhouse soil mix supplemented with Osmocote fertilizer and Micromax micronutrients). Keeping the growth chamber conditions maintained at day/night temperature (24/18 °C) and light/dark period (14 h/10 h), shoot height of wheat seedlings was measured at indicated timepoints. In subsequent experiments shoot and root lengths were measured at 24 and 48 hours after treatment described above (HAT) while growing in the plastic boxes in the dark. For each timepoint/treatment 16 – 18 seedlings were used for measurements.

### RNA extraction, RNA-seq analysis, RT-qPCR

To understand the underlying genetic variation resulting from the BMVE treatment, an RNA-seq study was conducted. BMVE treatment improved seed germination rate for both wheat and rice as early as 24 HAT (see Results) so we chose this time after treatment for transcriptome analysis of rice. Seeds were germinated and treated as described above. The three treatments used for total RNA extraction included a control minus BMVE (C24), BMVE treated (B24), and non-treated seeds harvested immediately after the 24 h of imbibition (C). Three biological replicates consisting of about 25 seedlings each were used for each treatment. At these stages, intact seeds were immediately frozen using liquid nitrogen. Following the collection, seed tissue was ground to a fine powder using liquid nitrogen with a mortar and pestle. Each finely ground tissue sample weighing ~100 mg was used for RNA extraction using Trizol-Phenol method as described in (Chomczynski and Mackey, 1995; Li and Trick, 2005). Upon isolation, RNA was purified using RNeasy MinElute cleanup kit (Qiagen) and subsequently treated with DNase. High quality RNA samples were sequenced in the University of Nebraska, Medical Center core facility (Omaha, NE). RNA libraries were sequenced using 150-bp single-end reads on an Illumina Novaseq 6000. Later, the bioinformatics tools with default settings were deployed including *Fastqc* to test the quality of the reads, *Trimmomatic* for trimming the adapters and removing low quality reads, *Tophat2* to align the RNA-seq reads to the reference genome, *Htseq* to create the read count matrix, and *EdgeR* (bioconductor package) for DEG analysis (Andrews, 2010; Bolger et al., 2014; Anders et al., 2015; Trapnell et al., 2009; Robinson et al., 2010). After obtaining the read counts, the genes expressing differentially between the control and BMVE treatment at 24 HAT were considered to have passed the statistical significance threshold of false discovery rate (fdr) < 0.05, and fold change (FC) greater than 2 or smaller than 0.5. This yielded a list of 474 genes. The read count ratio for specific genes of interest was calculated for control and BMVE treatment at 24 HAT using untreated seeds at 0 HAT as the base line level. These ratios were converted to the z-score values. Gene ontology enrichment analysis for the genes was conducted using bioinformatic tool, ShinyGO (http://ge-lab.org/go/) (Ge et al., 2020).

To confirm the RNAseq results as well as to gain temporal insights into the expression of certain genes, we performed real time quantitative PCR (RT-qPCR) assays. Treatment and stages that have been used for RT-qPCR assays included 2, 6, 12, and 24 HAT for control and BMVE treatment, respectively, along with non-treated uniformly pre-germinated seeds (24 hours after imbibition). SuperScript VILO cDNA synthesis kit (Invitrogen) was used to synthesize cDNA from 1µg of total RNA. RT-qPCR (10 µL) reaction mix prepared using gene-specific primers, SYBR Green Master Mix (Bio-Rad), and template were run on a Lightcycler 480 Real-Time PCR system (Roche). Ubiquitin (*UBQ5*) was used as a reference gene and endogenous control. Thermal cycling conditions were followed as described by Fragkostefanakis et al. (2015). Standard methods for data analysis were followed as described in Livak and Schmittgen (2001). In these RT-qPCR assays, a minimum of two independent biological replicates and three technical replicates were used.

### Ground-bed study

The ground-bed study was conducted to investigate if the growth enhancing effects of BMVE observed during germination and seedling growth were maintained during later vegetative growth stages. Wheat seeds were first treated with BMVE or control treatments at the seedling level as described above. Treated seeds were then grown in plastic cuvette trays filled with soil (greenhouse mix) until transplanted. After 7 days of growth in trays, treated and control seeds were transplanted into the ground bed (UNL Plant Growth Facilities, Lincoln, Nebraska). The ground-bed area was divided into two regions, one each for well-watered (WW) and water-limited (WL) conditions. In each region, 3 plots of 90x45 cm each, spaced at 20 cm, were randomly assigned to BMVE and control treatments (**Supplementary Figure 1A**). The spacing between the seedlings was set at 18x15 cm. Each plot had 24 wheat plants.

### Irrigation of the plots

The soil composition of the ground-bed was a silty clay loam. The calculated percentage of volumetric water content (VWC), field capacity (FC), and permanent wilting point (PWP) for this soil type were reported as 77.356 *SMP* ^−0.181^, 41.08%, and 20.588%, respectively. Plant available water (PAW) was calculated by subtracting PWP from FC which equates to 20.49%. Manageable allowable water depletion for this soil type was 35% of PAW depleted from FC which is 33.9%. It implies that severe drought stress begins when VWC dips lower than 33.9%. Surface drip pipes were used to water the plots throughout the experiment. To monitor the irrigation, water sensors were deployed at three depths (1, 2, and 3 feet) at two locations in each region. Volumetric water content was monitored every day and irrigation decisions were made accordingly. The ground-bed was located inside the greenhouse where the major source of water loss was evapotranspiration. Hence, it was difficult to impose complete drought conditions during the duration of the experiment. However, the water sensor data corresponding to WL plots indicated a declining trend for the VWC at 1 ft (**Supplementary Figure 1B**). Regularly applied irrigation to WW plots kept the VWC constant over time (**Supplementary Figure 1B**). Data from water sensors indicated that water requirements were met for WW plots whereas WL plots began experiencing drought at about 45 d.

### Phenotyping

For digital phenotyping, a recently developed aerial gantry imaging system was used to image the plots twice a week (Palli et al., 2019). The gantry imaging platform is an autonomous/semi-autonomous navigation system equipped with a trolley carrying camera suspended over the plots to capture crop canopy images (Palli et al., 2019). Imaging data were further processed in MATLAB to compute the pixel sum of the canopies. Later, the pixel sum was used as a proxy to characterize the aboveground biomass content of the plants. To complement the 2-D imaging data from the gantry system, manual measurements were taken alongside digital phenotyping once a week starting from week 2 to 9 after transplanting. These measurements include height (length of the longest leaf), number of tillers, and number of leaves. Due to the complexity in efficiently counting the large number of leaves, leaf counting was carried out only until 7 weeks after transplanting (WAT). At the end of week 9, plants were harvested to measure fresh weight following which the plants were oven dried at 70°C for 7 days to measure dry weight.

### BMVE rice panicle spray

The BMVE spray study was conducted to determine the effect of BMVE application during post-fertilization events on rice seed development and on germination in the next generation. As the rice seed developmental phase is very sensitive to high temperatures, we incorporated heat stress into the study to test if the BMVE treatment moderated the stress-mediated detrimental effects on seed development. Rice plants were grown in control conditions until flowering. During the onset of the flowering phase, panicles were carefully tracked and florets were marked upon the completion of fertilization (Dharni et al., 2022). Twenty-four hours after fertilization (HAF) of marked florets, subsets of plants were sprayed with BMVE or control solutions. To avoid spray drift, collar sheets were used around the panicles. Due to the highly sensitive nature of seeds to external treatments around the time of fertilization, the BMVE concentration used for spray was 0.1 mM instead of 0.5mM, which was used earlier for mature seed treatment. Immediately after the spray at 24 HAF, half of the plants for each treatment were kept in the control greenhouse (16 h light and 8 h dark at 28 ± 1°C and 23 ± 1°C) while the rest were moved to a greenhouse with high day and night temperatures (HDNT; 16 h light and 8 h dark at 36 ± 1°C and 32 ± 1°C). In total we had four treatment combinations; control treated/control environment, BMVE treated/control environment, control treated/HDNT environment, and BMVE treated/HDNT environment. A minimum of 3 plants per treatment combination was used and multiple seeds per plant were recovered. We first measured the fresh weight (FW) of developing seeds at 1, 2, and 4 days after treatment (DAT), and then measured the dry weight (DW) of seeds oven dried at 40 °C for 10 days.

Another set of similarly treated plants was grown to maturity under control conditions. Stressed counterparts of this set were also moved back to the control environment after being exposed to HDNT stress for 1, 2, and 4 d after treatment (DAT), respectively. These sets of plants were used to measure mature yield traits of marked seeds including mature seed weight, and morphometric parameters (length, width) for all four treatment combinations. For morphometric assessments, 50 marked seeds per treatment combination were first scanned with the WinRhizo scanner, and then analyzed using *SeedExtractor* (Zhu et al., 2021a). Later, we conducted germination assays using the harvested seeds from plants corresponding to all four treatment combinations. After surface sterilization, 45 seeds per treatment were germinated in 3 separate plastic boxes (as discussed above) and were considered germinated when their shoot length reached at least 0.2 cm. Following this criterion, seed germination was scored at 36, 60, and 84 hours after beginning of imbibition. Once ~99% germination was achieved both the root and shoot length of seedlings were measured using a ruler.

## Results

### Wheat seedling treatment with BMVE enhances early seedling growth

**Figure 1:**
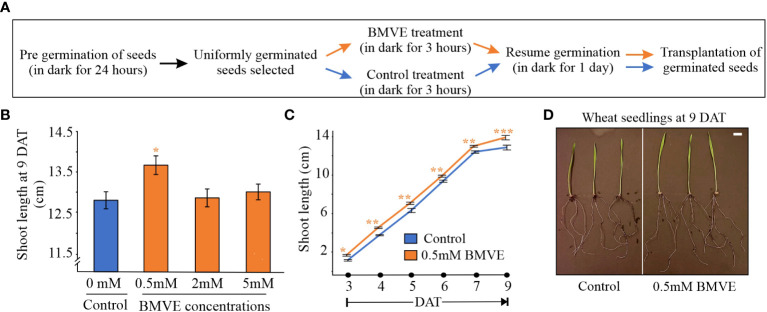
BMVE wheat seedling treatment. **(A)** Protocol for treating crop seeds with BMVE. **(B)** BMVE concentration effect on wheat shoot length 9 days after treatment (DAT). **(C)** Shoot length from 3 to 9 DAT. Statistical analysis is by Student**’**s t-test (α= 0.050); n = 16 per treatment/timepoint for shoot length data; ± bars represent standard error. Level of significances corresponding to p-value < 0.05, 0.01, or 0.001 are denoted by ‘*’, ‘**’, or ‘***’, respectively. Similar results were obtained from two independent experiments. **(D)** Representative wheat seedlings at 9 DAT. White bar represents 1 cm.

In earlier work we aimed to characterize the efficacy of one representative STA called BMVE (US9464283B2, Kamtec LLC) (Yokoyama et al., 2015). When mature dry wheat seeds were coated with BMVE we saw inconsistent results on the subsequent growth of plants in greenhouse studies (Singh, 2018). We hypothesized this may be due to inherent variability in the timing of seed germination among the relatively small number of seeds tested, and/or to suboptimal BMVE treatment conditions, either of which could obscure detection of potential beneficial effects. Therefore, we developed an improved protocol for wheat seed treatment that involved imbibing seeds for 24 h, selecting for uniformly germinated seeds (radical around 2 mm), followed by soaking these seeds in BMVE or control solution for 3 h. Growth was then continued for 24 h in the dark, followed by transplantation to soil and growth in the light (**Figure 1A**).

**Figure 2:**
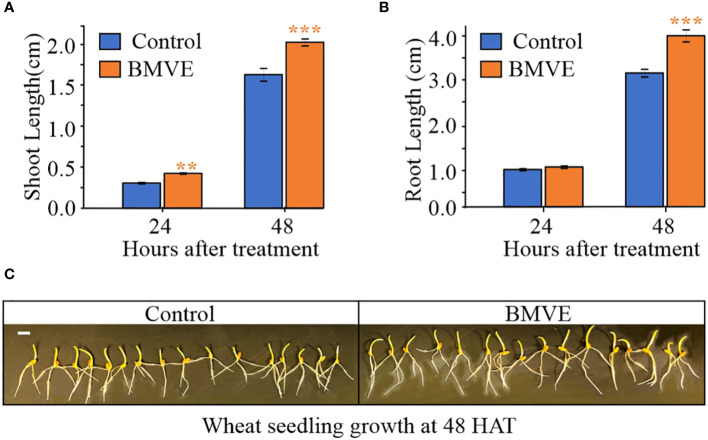
Effect of BMVE (0.5 mM) on early wheat seedling growth. Parts **(A)** and **(B)** represent average wheat shoot and primary root lengths at 24 and 48 HAT. For statistical analysis, a student**’**s t-test was conducted (α= 0.050); n = 16 to 20 per treatment/timepoint for shoot and root length data; ± bars represent standard error. Level of significances corresponding to p-value < 0.05, 0.01, or 0.001 are denoted by ‘*’, ‘**’, or ‘***’, respectively. **(C)** visual of germinating wheat seeds at 48 HAT. Horizontal white bar represents 1 cm.

To identify a BMVE concentration producing a positive effect, seeds were treated with 0, 0.5, 2 or 5mM BMVE, and shoot height was measured 9 days after treatment (DAT) following growth in soil. Greater shoot length was seen from the 0.5 mM BMVE treatment (p-value < 0.05), whereas higher concentrations were not effective (**Figures 1B, D**). We next determined whether positive BMVE effects were detectable at earlier times. A significant increase in shoot length was seen as early as 3 DAT and for each time point after that up to 9 DAT, when the maximum difference was observed (**Figure 1C**). To test for even earlier effects on both roots and shoots, treated seedlings were incubated on moist germination paper under dark conditions. A significant increase in shoot length was seen at 24 h after treatment (HAT) (**Figure 2A**). Primary root length enhancement was detected at 48 h after treatment (**Figure 2B**). **Figure 2C** shows a representative sample of wheat seedlings grown to 48 HAT. Similar results were obtained from two independent experiments.

### Seedling treatment confers tolerance to water limitation in mature plants

To learn whether the positive effect of BMVE on seedling growth translates to later stages of vegetative growth, a larger study with wheat was conducted in which control and BMVE treated wheat seedlings were transferred to a greenhouse ground bed. After 7 d of growth in soil, treated seedlings were transplanted to the ground-bed and growth was monitored using an overhead gantry imaging system as well as by manual phenotyping. Both well-watered (WW) and a water-limited (WL) stress condition were evaluated. Three plot repetitions were randomly assigned to each of the seed treatments (Control and BMVE) under each irrigation regime, and each plot contained 24 healthy wheat plants (**Supplementary Figure 1A**). Importantly, although water was withheld from the beginning for the WL treatment, a soil water deficit was not evident until around 34 days after transplanting (DATP) and did not reach the severe drought stage until about 51 DATP (**Supplementary Figure 1B**). 2-D imaging was carried out twice a week from 28 to 52 DATP. In previous studies, pixel sum has been used as a proxy to estimate the biomass content of the imaged plants (Chen et al, 2014.; Hartmann et al., 2011; Fahlgren et al., 2015; Zhu et al., 2021b).

**Figure 3:**
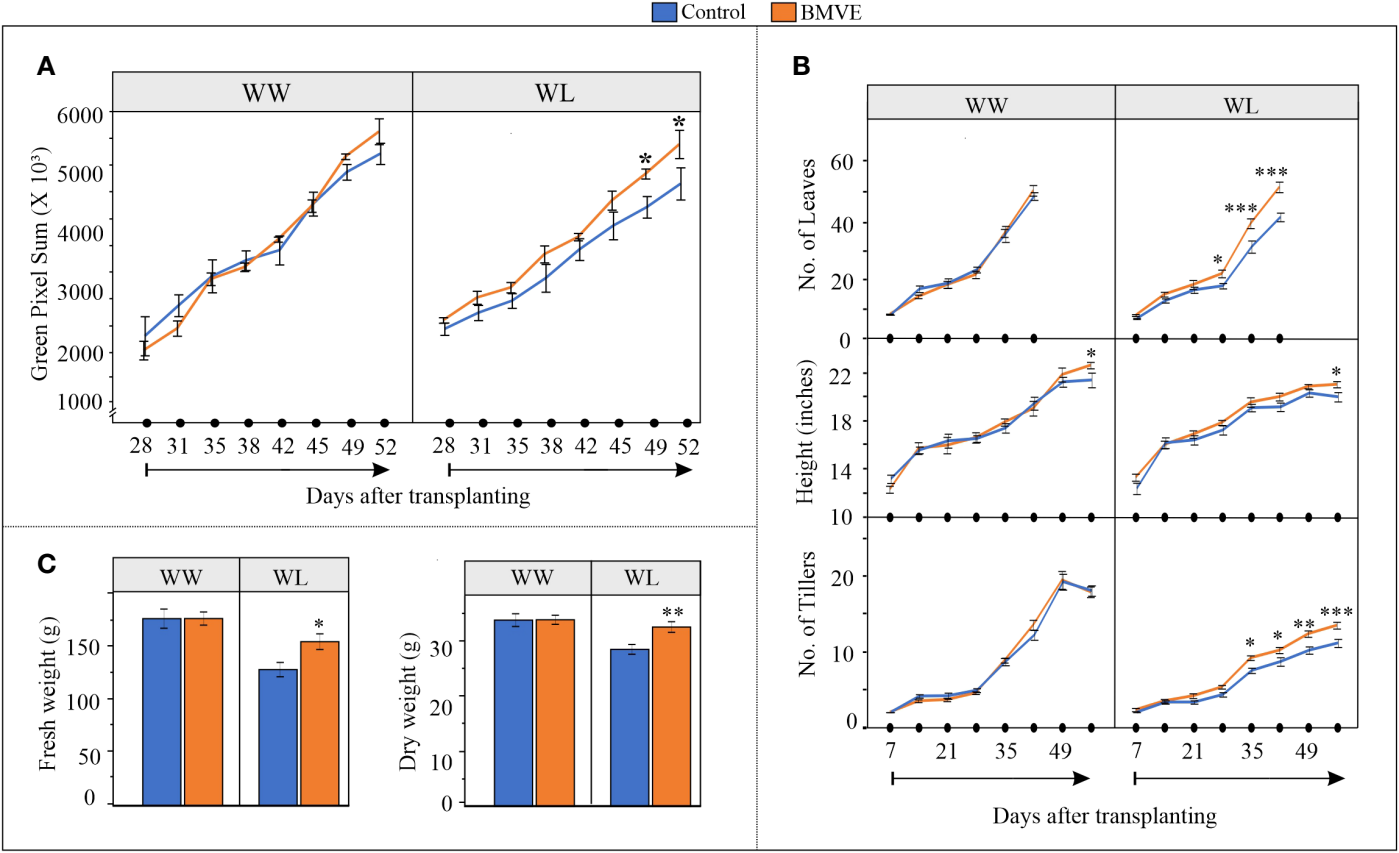
Impact of BMVE seed treatment on plants grown in a greenhouse ground-bed. **(A)** Digital imaging measurements captured by an automated gantry system were processed to calculate the aboveground green pixels. **(B)** Parameters documented manually during plant growth including number of leaves, plant height, and number of tillers. n = 12 per treatment/per water condition/per timepoint **(C)** Fresh and dry weights of the harvested above ground biomass at 56 days after transplanting. n = 16 per replicate/per treatment/per water conditions, and three replicates per treatment were used. For statistical analysis, a student’s t-test was conducted (α= 0.050); ± bars represent standard error. Level of significances corresponding to p-value < 0.05, 0.01, or 0.001 are denoted by ‘*’, ‘**’, or ‘***’, respectively.

Our results show that under WW condition there is no significant difference in pixel count for plants from the BMVE treatment versus the controls (**Figure 3A**). However, under WL conditions pixel count for BMVE treated plants was significantly higher than for control plants for days 49 and 52, time points when plants were experiencing severe drought (**Supplementary Figure 1**). Manual measurements gave similar results in that BMVE had no beneficial effect under WW conditions for any trait measured, with the exception that height was modestly higher for BMVE treatment at 52 DATP (**Figure 3B**). Under WL the number of leaves and tillers was significantly greater for BMVE treated plants beginning at 28 and 35 DAP, respectively. This is around the time when drought was becoming evident. Plant height was only greater at 52 d and counting of leaf number was not feasible after 42 DATP because of the large number of leaves by that time. End point measurement of fresh and dry weights at 52 DATP confirmed the results of the pixel count data analysis, yielding a significant difference for BMVE treatment only under WL conditions (**Figure 3C**). These results show that the positive BMVE effect on seedlings continued to older plants, but only when water deficit was apparent in the soil.

### BMVE enhances rice seedling growth

**Figure 4:**
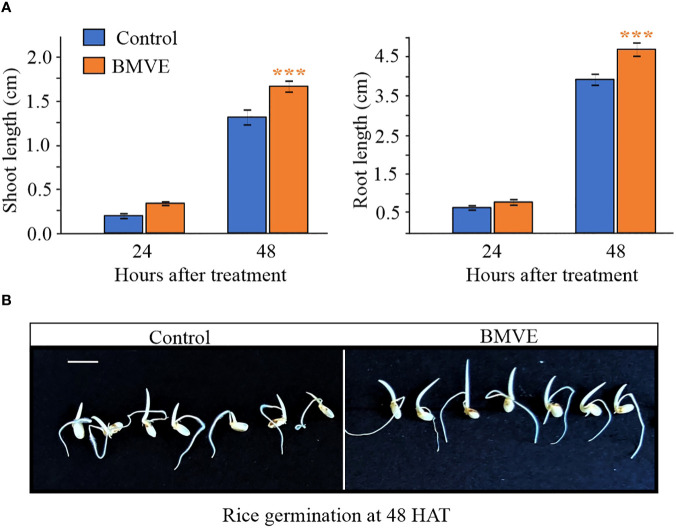
Growth of rice seedlings treated with BMVE. **(A)** Shoot and root lengths at 24 and 48 HAT. For statistical analysis, a student**’**s t-test was conducted (α= 0.050); n = 16 to 20 per treatment/timepoint for shoot and root length data; ± bars represent standard error. Level of significances corresponding to p-value < 0.05, 0.01, or 0.001 are denoted by ‘*’, ‘**’, or ‘***’, respectively. The experiment was repeated twice, and similar results were obtained. **(B)**representative germinating rice seeds at 48 HAT. Horizontal white bar represents 1 cm.

We next tested whether the wheat seedling treatment protocol yielded similar results in rice. Rice seedlings treated with BMVE (0.5mM) also exhibited significantly higher shoot and root length at 48 HAT (**Figure 4A**). At 24 HAT shoot and root length were not significantly higher for BMVE treated rice seedlings. Representative rice seedlings at 48 HAT are shown in (**Figure 4B**). Two independent experiments gave similar results, showing that BMVE enhances seedling growth in at least two major crops.

### BMVE alters the transcriptome of rice seedlings

We hypothesized that transcriptome changes after BMVE treatment of seedlings would precede the first noticeable enhancement of shoot and root growth at 48 HAT. To capture these early alterations, RNA-seq analysis was performed on rice seedlings corresponding to the 24 HAT growth stage for both the control and BMVE treatment, which we refer to as C24 and B24, respectively. Seedlings that were only imbibed for 24 h and not treated with BMVE were also sampled at that time point (C0) to decipher the differential temporal changes in gene expression that result from BMVE treatment. A total of 472 differentially expressed genes were identified between B24 and C24 (FDR adjusted p-value 0.05). Among the 472 total genes, 194 showed increased transcript level (FC > 2) while 278 genes showed decreased transcripts (FC < 0.5).

**Figure 5:**
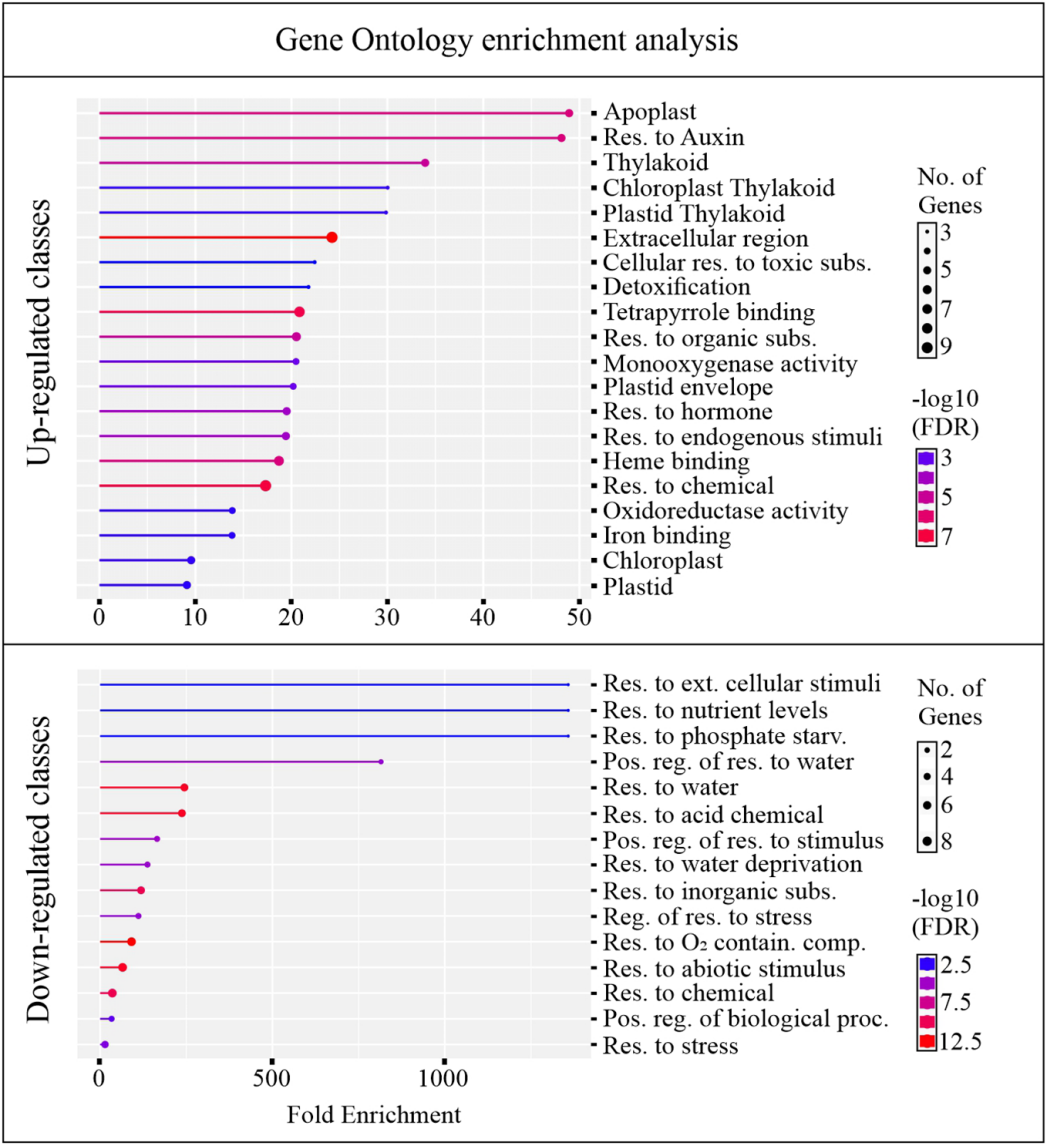
Gene ontology (GO) enrichment. The analysis was conducted for genes differently regulating between BMVE, and control treated rice seeds at 24 HAT. The genes passing the statistical threshold of p-value < 0.05, false discovery rate (fdr) < 0.05, and fold change greater or less than 2 were considered for this analysis.

An overview of altered biological pathways between C24 and B24 was obtained through gene ontology (GO) enrichment analysis. This yielded 22 and 10 unique genes for upregulated and downregulated classes, respectively, with most genes falling within multiple GO terms (**Supplementary Table 1**). About 75% of the up-regulated genes were overrepresented among 20 GO terms, with enrichment ranging from about 9 to 48-fold (**Figure 5**). Most of the enriched GO terms in the upregulated category were associated with detoxifying processes, responses to toxic substances, development, and hormonal activity. On the other hand, about 20% of downregulated genes were grouped into 15 GO terms with enrichment ranging from around 1 to 1300-fold. Largely, these downregulated GO terms were associated with stress responses including abiotic, biotic, and water deprivation (**Figure 5**).

### Transcripts associated with reducing ROS load are elevated by BMVE

**Figure 6:**
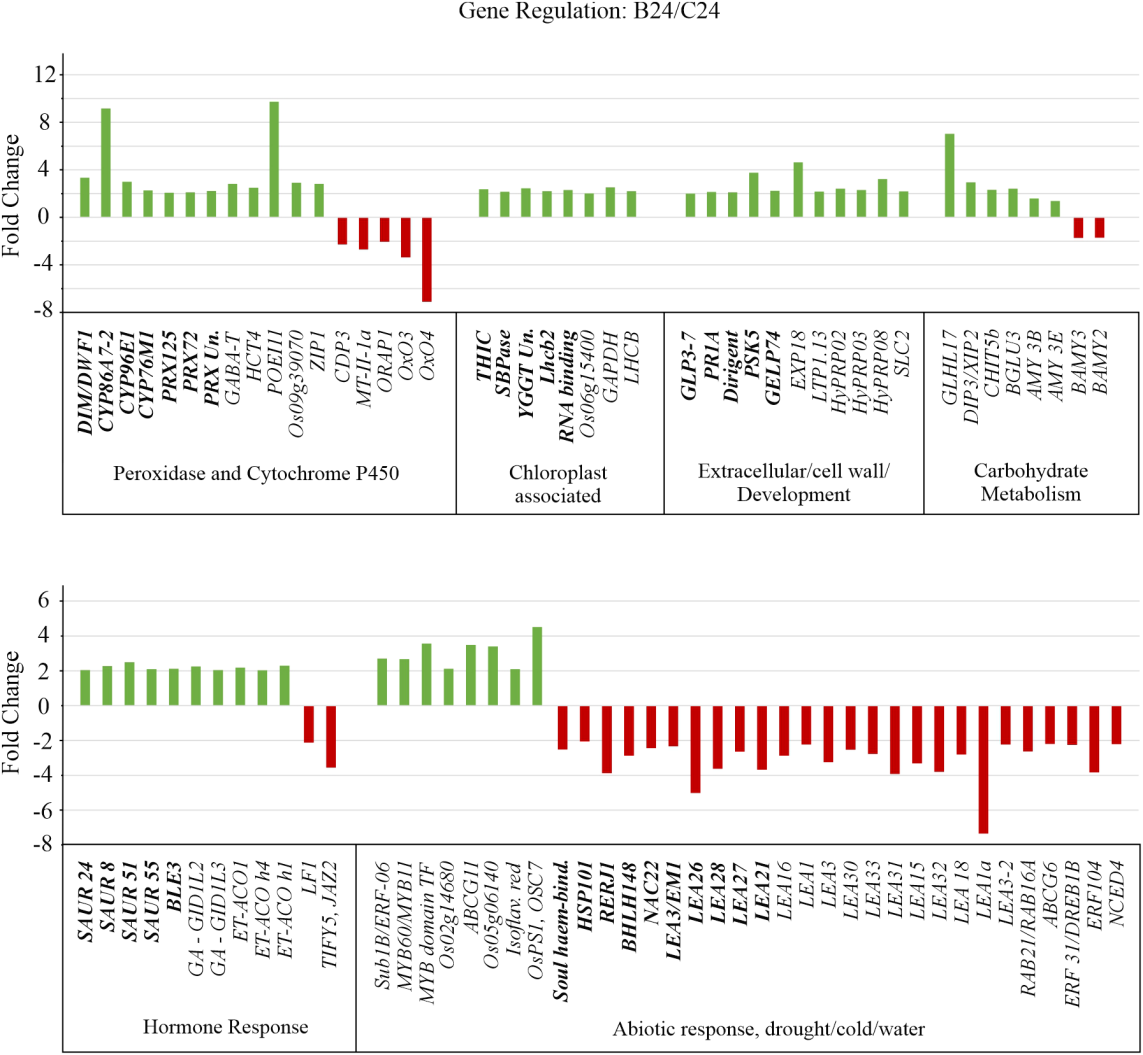
Gene expression altered by BMVE treatment in comparison to control treatment at 24 HAT. Transcript differences with p-value < 0.05, false discovery rate (fdr) < 0.05, and > 2-fold change were included in the analysis. Unique genes recovered from Gene Ontology (GO) enrichment analysis are shown in bold, additional differentially regulated gene shown in plain text.

GO term enrichment revealed that classes corresponding to ‘cellular response to toxic substances, detoxification, monooxygenase activity, and oxidoreductase activity’ were enriched with genes exhibiting increased transcript abundance (**Figure 5**). **Figure 6** shows the FC of genes discovered by GO enrichment (labeled in bold). Rapid breakdown and oxidation of nutrient reserves during seed germination results in accumulation of reactive oxygen species (ROS) that can delay or prevent seedling growth (Kurek et al., 2019; Thakur and Vasudevan, 2019). Activation of antioxidant systems such as peroxidases (PRXs), catalases (CATs), and superoxide dismutases (SODs) facilitate ROS removal and counteract potential molecular damage (Dietz et al., 2016; Steinbrecher and Leubner-Metzger, 2017; Noctor et al., 2018; Kurek et al., 2019; Marks et al., 2022). Several peroxidases (PRXs) and Cytochrome P450 genes appeared in our GO analysis results (**Supplementary Table 1**) and these are generally known to be involved in H_2_O_2_ metabolism and enhancing the activity of compounds with increased antioxidant activity (Almagro et al., 2009; Pandian et al., 2020; Rao et al., 2020; Zhang et al., 2022). Other upregulated categories in the GO analysis included apoplast, chloroplast, extracellular region, heme binding, plastid, response to hormone, and response to chemical (**Figure 5**). These classes include genes such as *DIM/DWF1 (LOC_Os04g48200), Dirigent (LOC_Os11g42500), OsGLP3-3 (LOC_Os03g58980), OsLhcb2 (LOC_Os03g39610)*, and *OsPSK5 (LOC_Os03g47230)* that are important for maintaining antioxidant systems and regulating ROS production (Thamil Arasan et al., 2013; Han J. et al., 2014; Li et al., 2016; Nahirñak et al., 2019; Jiang et al., 2021; Yang et al., 2006) (**Supplementary Table 1**).

**Figure 7:**
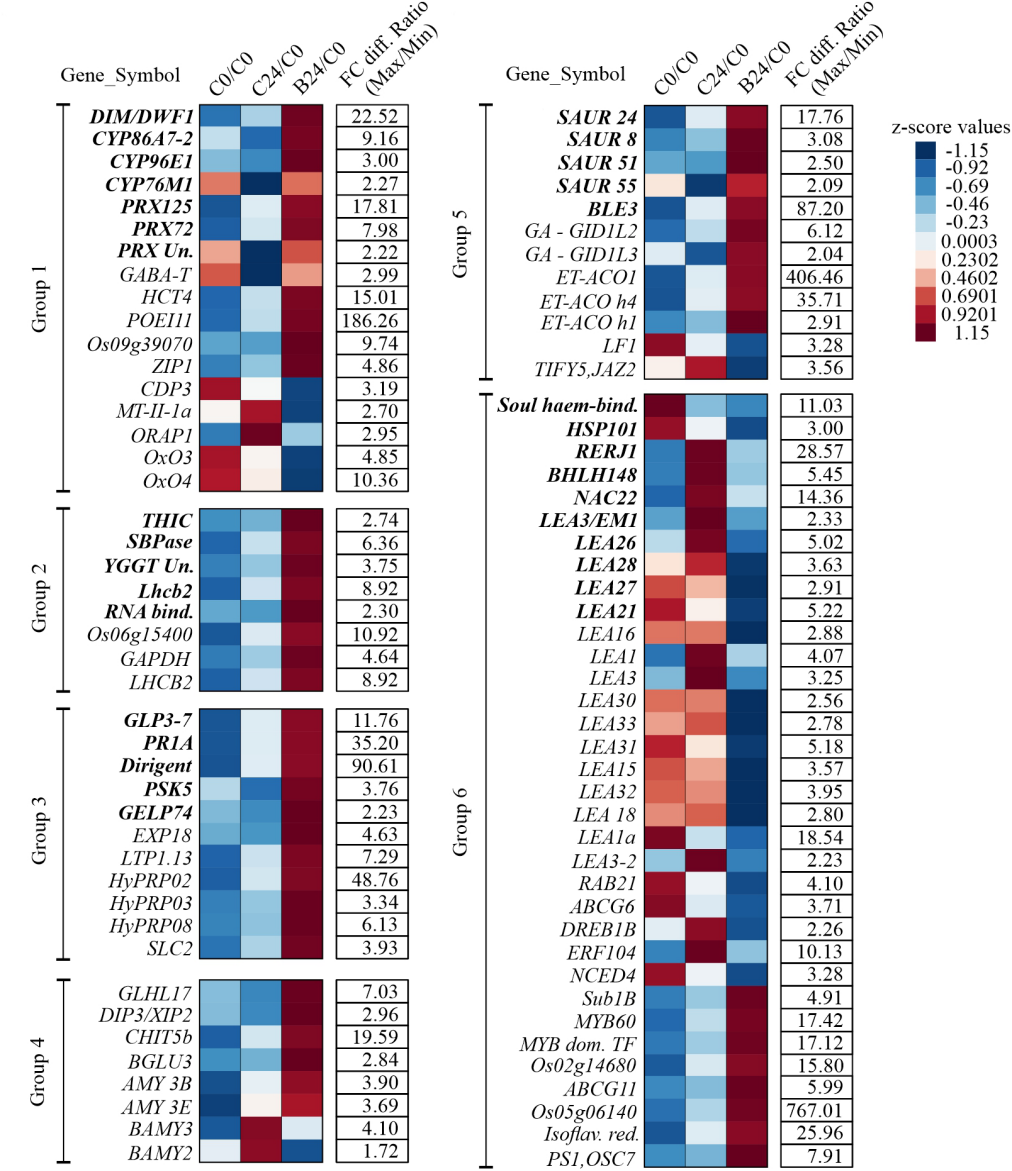
Temporal expression of the genes for C24 and B24 in comparison to untreated seeds (C0). Magnitude of the expression differences is depicted by z-score of the ratios calculated by dividing the average transcript reads for both the treatments at 24 HAT with the read count for untreated control seeds. Fold change differences in the ratios between maximum and minimum values per row are provided in the column ‘FC diff. Ratio (Max/Min)’. ‘C0’, ‘C24’, and ‘BM24’ represent untreated control, control at 24 HAT and BMVE treatment at 24 HAT, respectively. Unique genes recovered from Gene Ontology (GO) enrichment analysis are shown in bold fonts, additional differentially regulated genes are in plain text. Genes representing common functions are clustered together into six groups. Group 1: Peroxidases, cytochrome P450s, and detoxification associated genes; Group 2: Chloroplast and thylakoid associated genes; Group 3: Extracellular region, cell wall, and development associated genes; Group 4: Carbohydrate (CHO) metabolism; Group 5: Hormonal response; Group 6: Response to stress (abiotic, biotic, water, and chemical).

To assess how BMVE perturbs the normal temporal changes in early seedling transcripts. **Figure 7** shows transcript abundance for control and BMVE treatment compared to C0, expressed as z-scores. For example, PRX125 transcript normally increases over 24 h of seedling growth (C0/C0 vs. C24/C0), but BMVE treatment enhances the increase (B24/C0). On the other hand, the expression of some Cytochrome P450s (*CYP86A7-2*, *CYP96E*, *CYP76M1*), PRX (*LOC_Os04g51300*), and *PSK5* is reduced in seedlings from C0 to C24, but BMVE treatment either increases or maintains the expression of these transcripts over the first 24 h (**Figure 7**). Overall, these results suggest that one effect of BMVE treatment is to help reduce the ROS load, allowing for more vigorous seedling growth at this stage.

### Transcripts associated with cell elongation and growth are elevated by BMVE

Other genes identified by GO analysis in the upregulated classes include four small auxins up RNAs (*SAUR*) *8, 24, 51*, and *55* (**Figures 6**, **7**, **Supplementary Table 1**). Although their function is unclear, *SAUR* genes respond quickly to exogenous and endogenous auxin and they are expressed predominantly in developing tissues, especially the elongating hypocotyl (Gee et al., 1991; Jain et al., 2006). *OsBLE3* (*LOC_Os05g15630*) is another upregulated auxin responsive gene reported that was reported to be involved in cell elongation in rice through dual regulation by brassinolide and auxin (Yang et al., 2006). The Calvin-Benson cycle gene, *SBPase* (*LOC_Os04g16680*), was also upregulated for B24. Mutation in this gene results in decreased chlorophyll content during the seedling stage in rice (Li et al., 2020). Additionally, *SBPase* is known to be upregulated by chitosan treatment, which positively impacts plant carbon metabolism and photosynthetic components ultimately leading to enhanced plant growth and development (Chamnanmanoontham et al., 2015). Consistent with the physiological observation, several genes associated with growth are up regulated by BMVE treatment.

### Several transcripts associated with abiotic stress are reduced by BMVE

Ten unique down-regulated genes were grouped by GO enrichment into abiotic stress response categories. Most of the genes in this category normally exhibit higher expression over time (**Figure 7**, C0 vs. C24). However, for B24 their transcript levels are significantly less as compared to C24. The abscisic acid (ABA) inducible genes *OsNAC22 (LOC_Os03g04070), Osbhlh148 (LOC_Os03g53020)*, and *LEA26 (LOC_Os11g26750)* are known to modulate stress tolerance responses through ABA dependent signaling pathways (Seo et al., 2011; Hong et al., 2016; Verma et al., 2017). *OsEm1/OsLEA3 (LOC_Os05g28210)* encodes an ABA regulated small hydrophilic plant seed protein whose expression is reduced in the embryos dissected from germinating seeds and co-expresses with the genes belonging to *LEA* or dehydrin protein family (Hattori et al., 1995; Han C. et al., 2014). In addition to *OsEm1*, all other *LEA 21, 26, 27, and 28* genes exhibited decreased expression in BMVE treated seeds at 24 HAT (**Figure 7**). *OsRERJ1* is involved in rice shoot growth inhibition mediated by jasmonic acid (Kiribuchi et al., 2004), and its expression was also reduced under BMVE treatment. In summary, BMVE treatment shifted the normal trajectory of many transcripts by increasing levels for genes involved in antioxidant activity and auxin/growth responses, while reducing transcripts for abiotic stress response.

### BMVE modulates additional genes involved in seed germination processes

To broaden the scope of the analysis we identified 58 additional genes not identified by GO analysis that were nevertheless significantly affected by BMVE and were associated with the major GO classes (shown as regular font in **Figures 6**, **7**). These belonged to the classes including oxidoreductase activity, detoxification, cell wall development, carbohydrate metabolism, hormone, and response to abiotic/biotic stresses (**Figures 6**, **7**).

Among these are three genes, *GABA-T* (*LOC_Os02g02210*), *HCT4* (*LOC_Os06g08640*), and *POEI11* (*LOC_Os10g05950*) that are involved in monooxygenase and oxidoreductase activity. *HCT4* and *POEI11* normally increase over time, while *GABA-T* declines (C0 vs. C24), but in each case BMVE treatment resulted in higher transcript levels (C24 vs. B24). (Wu et al., 2006; Kim et al., 2012; You et al., 2014). Activity of *GABA-T* defends against environmental stress by preventing cell death and restricting the accumulation of reactive oxygen intermediates (Wu et al., 2006). *HCT4* is involved in catalyzing the biosynthesis of cinnamic acid conjugates that function as antioxidants (Zhao and Moghadasian, 2010; Kim et al., 2012). Expression of two heavy metal detoxifying genes i.e., *LOC_Os09g39070* and *ZIP1*, involved in aluminum, zinc, cadmium, and copper stress tolerance mechanisms in plants was also increased for B24 (Wang et al., 2014; Liu et al., 2019). In contrast, the expression of the genes (*CDP3*, *MT-II-1a, ORAP1*, *OxO3*, and *OxO4*) that are either induced by or promote production of hydrogen peroxide was reduced for BMVE treated seeds (Hu et al., 2003; L. Zhou et al., 2005; Ueda et al., 2015; Peng et al., 2022). Over time, *CDP3*, *OXO3*, and *OXO4* exhibited a slight decline in transcript levels for control seeds, however BMVE treatment led to even greater reductions (**Figure 7**).

Hormonal signaling plays a vital role during germination and early seedling growth (Ni et al., 2001; Seo et al., 2009; Ren and Gray, 2015; Sun et al., 2020). In addition to the SAUR class genes mentioned earlier, our analysis revealed additional differentially genes. Genes involved in ethylene biosynthesis, such as 1-aminocyclopropane-1-carboxylate oxidase *ACO1* (*LOC_Os09g27820*)*, ACO homolog 1* (*LOC_Os08g30100*)*, & ACO homolog 4* (*LOC_Os08g30080*), were upregulated for BMVE treated seeds at 24 HAT (**Figure 6**). In contrast, ethylene response factors *ERF-104* (*LOC_Os08g36920*) and *ERF3*/*DREB1b* (labeled *DREB1B* in **Figure 7**) (*LOC_Os09g35010*) are normally elevated as seedling development progresses (C0 vs. C24) (**Figure 7**), but BMVE essentially eliminates the increase during this time (B24). *ERF-104* is associated with cold stress response (Li et al., 2018) and *ERF3*/*DREB1b* are associated with cold and drought. This is consistent with the reduction of several other stress associated transcripts mentioned earlier. On the other hand, an *APETALA2* transcription factor *ERF-063*, commonly known as *Sub1b* showed higher transcript levels for BMVE treated seeds at 24 HAT (**Figure 7**).

The 9-cis-epoxycarotenoid dioxygenase (*OsNCED4*; *LOC_Os07g05940*), a major gene involved in ABA biosynthesis, normally declines during germination and in our experiment the reduction is evident from C0 to C24 (**Figure 7**). However, BMVE treatment results in an even greater reduction in its transcript levels. During the process of seed germination an antagonistic reaction occurs between abscisic acid (ABA) and gibberellic acid (GA), where the former is known to repress germination (Müller et al., 2006; Golldack et al., 2013; Liu et al., 2016; Liu and Hou, 2018). *OsLF1* (*LOC_Os01g51610*)*, a B3 DNA* binding domain-containing protein that negatively regulates GA metabolism and promotes the ABA biosynthesis (Guo et al., 2013) also shows this pattern of enhanced downregulation by BMVE (**Figure 7**). Another ABA inducible gene that is involved in jasmonic acid signaling i.e., *TIFY5/JAZ2* (*LOC_Os07g05830*) (Ye et al., 2009) showed reduced transcript levels for BMVE treated seeds (**Figure 7**). On the other hand, two gibberellic acid receptors, *GID1L2* (*LOC_Os07g06860*) and *GID1L3* (*LOC_Os01g06220*) showed increased transcript abundance for BMVE treated seeds at 24 HAT (**Figure 7**) (Hartweck and Olszewski, 2006).

All of the differentially expressed *LEA* genes (including *LEA 3, 21, 26, 27*, and *28* from the GO enrichment analysis) showed reduced transcript levels for BMVE treated seeds at 24 h (**Figures 6**, **7**). Their expression either increases or slightly decreases over time for control seeds. *LEA* genes are normally expressed in response to ABA, dehydration, and salt stress, reinforcing our observation that in general, stress-related transcripts are reduced by BMVE treatment (Chandler and Robertson, 1994; Bray, 1997; Shinozaki, 2000; Wang et al., 2007).

Exceptions to this trend are genes involved in salt stress (*ABCG11*: *LOC_Os05g02890*; *LOC_Os05g06140*) and biotic stress (*Isoflavone reductase*: *LOC_Os01g01650*; *PS1/OSC7*: *LOC_Os11g08569*) tolerance, which showed upregulation for B24 (**Figure 7**). *ABCG11* is highly responsive to salt stress in rice, and its deficiency reduces the lipid metabolism in roots which further impact suberin biosynthesis (Panikashvili et al., 2010; Matsuda et al., 2012). A study comparing the difference in salt tolerance between barley and rice reported a higher expression of *LOC_Os05g06140* in the salt tolerant barley accession ‘XZ26’ (Fu et al., 2019). *Isoflavone reductase* upregulates in rice to defend against bacterial leaf blight disease and *PS1/OSC7* is a member of the gene family ‘2,3-oxidosqualene cyclase’ which catalyzes the first step in triterpene biosynthesis required for protection against pathogen and pests (Thimmappa et al., 2014; Peng et al., 2015; Xue et al., 2018).

Studies have shown that expansins operate as cell wall loosening proteins and are known to mediate coleoptile elongation under anoxic conditions (Cho and Kende, 1997; Lee et al., 2001; Magneschi et al., 2009). One such gene is the *OsEXP 18* (*LOC_Os03g06040*) gene from the ‘Expansin’ family and showed higher transcript levels for B24 (**Figure 7**). *SLC2* (*LOC_Os09g39720*) plays an important role in shoot development and salicylic acid production in rice (Liu et al., 2020). The expression of this gene normally increases over time (C24 versus C0), however, it exhibits even higher levels for BMVE treated seeds indicative of growth advancement (**Figures 6**, **7**). Three ‘PS Calvin cycle’ related genes, ‘*Glyceraldehyde-3-phosphate dehydrogenase* (*GAPDH; LOC_Os04g38600*)*, LOC_Os01g73540, and chlorophyll A-B binding protein* (*LOC_Os03g39610*)*’*, also showed higher transcript abundance for BMVE treated seeds (**Figure 7**). The upregulation of various growth associated genes for BMVE treatment at 24 HAT is consistent with the advancement in seedling growth observed at 48 HAT.

### BMVE modulates transcript abundance of carbohydrate metabolism genes

According to the triphasic model for seed germination, the TCA cycle or aerobic respiration is usually recovered during phase III at 50 hours after imbibition (Bewley, 1997). This phase is marked by the initiation of rapid cell division and increased energy demand resulting in increased activity of α-amylases following the breakdown of starch reserves (He and Yang, 2013). The induction of α-amylases during the initial stages of seed germination is regulated by ethylene (ET) and gibberellic acid (GA) (Sun et al., 2020). In general, the activity of ET and GA during seed germination promotes the expression of α-amylases while impeding the β-amylases (Sun et al., 2020). To explore further the changes in energy status among the rapidly (BMVE treated) and slow growing (control treated) seeds, we searched for all the DEGs related to starch metabolism. Our results indicated that two each of α and β-amylases consistently exhibited higher (*AMY3B: LOC_Os09g28420*, and *AMY3E: LOC_Os08g36900*) and lower (*BAMY2: LOC_Os03g04770*, and *BAMY3: LOC_Os10g41550*) expression trends (**Figure 7**). Additionally, the genes belong to *Glycosyl Hydrolase* family including *BGLU3: LOC_Os01g59840, CHIT5b: LOC_Os11g27400, DIP3/XIP2: LOC_Os05g15850*, and *GLHL17: LOC_Os01g71380* have higher transcript levels for BMVE treated seeds (**Figure 6**). The upregulation of amylase and glycosyl hydrolase genes is further evidence that seedling growth is advanced by BMVE treatment of seedlings.

### Evidence for earlier transcriptional changes

**Figure 8:**
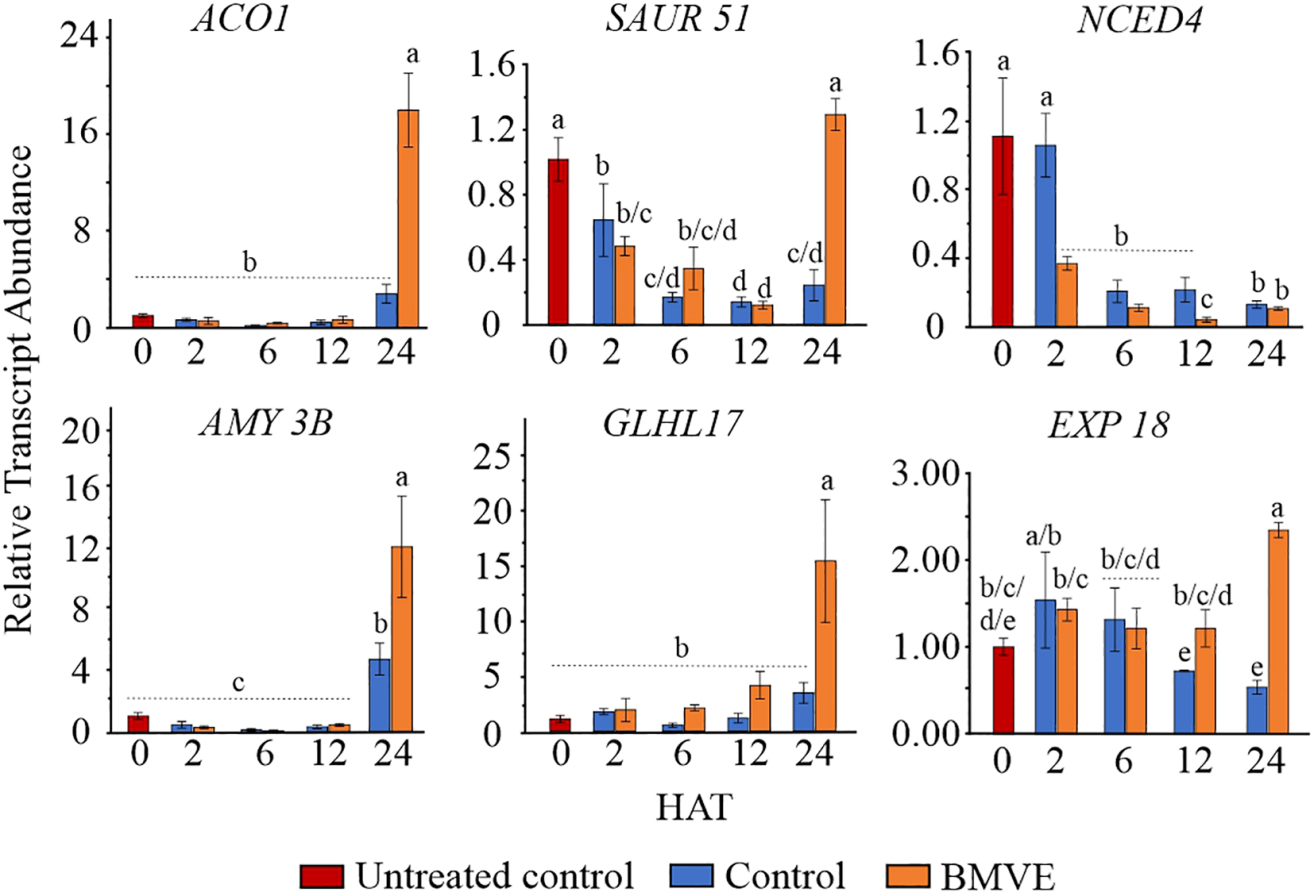
Gene expression analysis by real time quantitative PCR (RT-qPCR). At 2, 6, 12, and 24 hours after treatment (HAT) for both control and BMVE treatments, the relative transcript abundance of these genes was calculated against the transcript abundance of untreated control seeds at 0 HAT. These genes included aminocyclopropane-1-carboxylate (ACO1), small auxin up RNA (SAUR51), 9-cis-epoxycarotenoid dioxygenase (NCED4), α-amylase (AMY3B), glycosyl hydrolase family protein (GLHL17), and an expansin family protein (EXP18). For statistical analysis, a student**’**s t-test was conducted (α= 0.050); ± bars represent the standard error; values not connected by same letter are significantly different.

Transcriptome data for 24 HAT indicates that BMVE-induced acceleration of seedling growth correlates with up-regulation of growth-related hormonal genes and transcription factors, starch regulatory genes, and enzymes involved in anaerobic pathways. To validate these results and test whether BMVE stimulated genes prior to 24 HAT, six were evaluated by qPCR at 2, 6, 12 and 24 HAT (**Figure 8**). The results for 24 h aligned closely with the transcriptomic results, validating the overall accuracy of whole transcriptome data. BMVE increased the transcript abundance of genes associated with ethylene biosynthesis (*ACO 1)*, cell wall loosening (*EXP 18*), an auxin responsive factor (*SAUR 51*), a glycosyl hydrolase gene that degrades the callose deposited in the grain cell wall (*GLHL17*), and a major carbohydrate (CHO) metabolism gene (*AMY 3B*). Five of the upregulated transcripts showed no increase prior to 24 HAT. The exception was *EXP18*, which was significantly elevated relative to the control at 12 HAT.

In contrast, NCED was negatively affected by BMVE as early as 2 HAT. At 24 HAT qPCR indicated no difference for this treatment from BMVE treatment, although whole transcriptome analysis suggested a roughly 2-fold decline at 24 HAT. ABA activity negatively regulates the process of seed germination during very earlier stages and the ABA synthesis enzyme *NCED4* normally declines during germination (**Figure 7**) (Huo et al., 2013). Our results showing that BMVE treatment accelerates the early loss of *NCED4* transcripts suggests that early hormonal changes may regulate later gene and metabolic responses that are associated with growth.

### BMVE rescues rice seed grain parameters under heat stress

Embryo and endosperm development are sensitive to high temperature, which can negatively impact seed viability, germination, and seedling growth in the next generation (Warner and Erwin, 2005; Jagadish et al., 2007; Arshad et al., 2017; Paul et al., 2020). We investigated whether BMVE application to rice florets shortly after fertilization impacts development of the resulting seeds that were then exposed to heat stress during the embryo/endosperm development phase.

**Figure 9:**
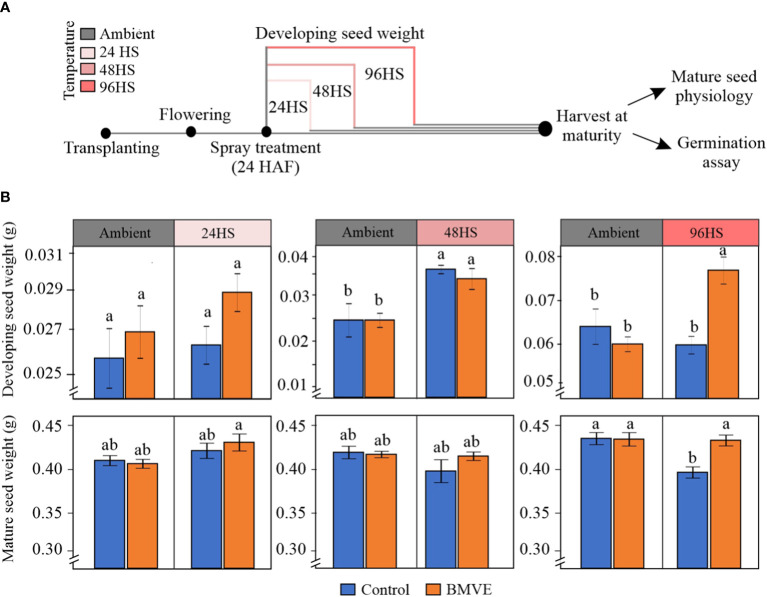
Effect of BMVE treatment on the rice grain development under heat stress (HS) conditions. **(A)** pictogram illustrating heat stress regime. Florets were marked at the time of fertilization, and 24 hours after fertilization (HAF) spray treatments were applied (BMVE: 0.1 mM and control). Following the spray, plants subjected to ambient (28 ± 1 °C and 23 ± 1 °C, light: dark) and heat stress (HDNT; 36 ± 1°C and 32 ± 1°C, light: dark) conditions. These plants were evaluated for developing seed weights at 24, 48, and 96 hours after the heat stress along with respective controls. Additionally, separate sets of plants were moved back to ambient conditions upon the completion of each type of stress regime and subjected to grow until maturity facilitating mature seed data collection. **(B)** shows the developing and mature seed weights from the plants that received above-mentioned treatments. For statistical analysis, a student**’**s t-test was conducted (α= 0.050); n = 40 – 50 seeds per treatment/timepoint from 8 replicates (developing grain weight) and 60 – 80 seeds per treatment from 8 replicates (mature grain weight); ± bars represent the standard error; values not connected by same letter are significantly different. The whole experiment was repeated twice.

We marked recently fertilized florets and then whole panicles were sprayed 24 h later with BMVE (100 uM) or control solution (Dhatt et al., 2021; Dharni et al., 2022). Plants were then moved to control (28 ± 1 °C and 23 ± 1 °C light and dark, respectively) or high day and night temperature (HDNT; 36 ± 1°C and 32 ± 1°C light and dark, respectively) (**Figure 9A**). To avoid the more damaging impacts of severe heat stress, we chose a moderate stress regime following the BMVE treatment (**Figure 9A**). Plants were heat stressed for 24, 48 or 96 h corresponding to 48, 72 and 120 HAF, respectively. Developing seed weight for 24 and 48 HS showed non-significant differences between control and BMVE treated seeds under both ambient and HDNT conditions (**Figure 9B**). Developing seed weight for 48 HS was significantly higher than non-heat stressed seeds for both the control and BMVE treatments, probably due to precocious cellularization under this moderate level of heat stress (Chen et al., 2016; Begcy et al., 2018). For plants exposed to a 96 h of HDNT, BMVE treatment led to significantly higher FW of developing seeds. The experiment was repeated twice, and similar results were obtained.

Separate sets of similarly treated plants were grown to maturity to assess mature grain seed traits including weight and morphometrics of the marked grains. These plants had been moved back to control conditions after exposing them to the indicated HDNT regimes (**Figure 9A**). Mature grain weights were not significantly different for any treatment for 24 and 48 HS (**Figure 9B**). In contrast with fresh weight at 96 h, mature grain weight from control plants was negatively affected following the 96 HS treatment. However, BMVE treatment of these developing seeds restored the mature seed weight to that of non-heat stressed plants. This shows that BMVE treatment negates the detrimental impact of the 96 h HS imposed at the time of early seed development.

As a positive effect of BMVE treatment was only seen for 96 h HS, mature seeds from this time were used to conduct morphometric analysis. Grain length, width, and area are the major determinants of grain size and quality parameters, and contribute to final grain yield (Tan et al., 2000; Huang et al., 2013). BMVE treatment of developing seeds led to significantly greater grain length, in both ambient and heat stress conditions (**Table 1**). Grain width and area were unaffected by BMVE for the seeds developed under ambient conditions. However, the reduction in width caused by HS was restored to ambient level by BMVE, and grain area was significantly greater under HS for BMVE treatment compared with control. These results are consistent with the increased mature seed weight seen for BMVE treated seeds that experienced the 96 HS treatment.

**Table 1:** Grain morphometrics data of mature grains from ambient temperature-control spray, ambient temperature-BMVE spray, heat stress-control spray, and heat stress-BMVE spray conditions.

Temperature	Ambient		Heat stress	
Spray	Control	BMVE	Control	BMVE
Grain length	4.87 ± 0.03^b^	5.01 ± 0.03^a^	4.86 ± 0.03^b^	5.05 ± 0.03^a^
Grain width	2.96 ± 0.02^a^	3.00 ± 0.02^a^	2.89 ± 0.02^c^	3.01 ± 0.02^a^
Grain area	11.41 ± 0.13^b^	11.72 ± 0.12^ab^	11.02 ± 0.13^c^	11.78 ± 0.12^a^

For statistical analysis, a student**’**s t-test was conducted (α= 0.050) for each morphometric parameter individually; n=50-60 marked seeds; ± represents the standard deviation; values not connected by same letter are significantly different.

### BMVE treatment of developing seeds enhances germination

**Figure 10:**
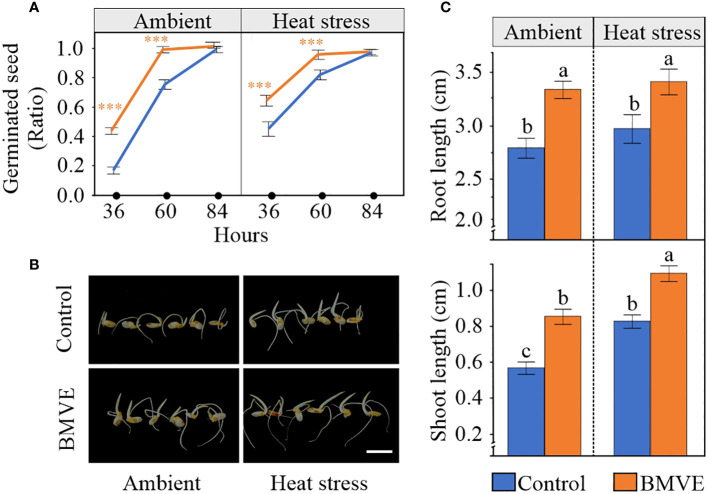
Effect of BMVE treatment of developing rice seeds in the next-generation. **(A)** Germination ratio for seeds from ambient and 96 h HS at 36, 60, and 84 hours after germination (HAG). **(B)** Representative rice seed germination at 84 HAG. Horizontal white bar represents 1 cm. **(C)** Root and shoot lengths at 84 HAG,for seeds germinated following the indicated treatment and temperature conditions. Statistical analysis was a student**’**s t-test (α= 0.050); n = 46 to 48 seeds per treatment; ± represents the standard error. Level of significances corresponding to p-value < 0.001 is indicated by ***.

We next tested whether the enhanced seed parameters resulting from BMVE treatment translated to increased seed germination and seedling vigor in the next generation. Seeds were considered to have germinated when the radicle emerged and the plumule reached the length of at least 0.2 cm. Seeds that developed following the BMVE treatment germinated faster than the untreated seeds from the heat stressed plants, correlating with the enhanced values for all parameters that were measured for these seeds (**Figure 10A**). Although grain length was the only morphometric trait affected by BMVE for non-heat stressed plants, BMVE treatment also enhanced germination of these seeds. For both temperature conditions BMVE treated seeds achieved more than 90% of germination by 60 hours whereas control treated seeds achieved only about 70% germination by that time. By 84 h all the seeds under observation were germinated, indicating the HS treatment was not severe enough to affect viability. Also evident is that this moderate HS treatment increased germination rate for both the control and BMVE treatments, consistent with previous findings (Begcy et al., 2018).

As expected from the faster germination rate for seeds that developed on BMVE treated plants, both the shoot and root length of these seedlings were significantly higher than the controls at 84 h after germination (**Figures 10B, C**). For the non-BMVE seeds, the shoot length was significantly higher for seeds that developed under HS as compared to ambient conditions while no differences were observed for root length. This is consistent with previous findings that moderate HS during the seed development phase accelerates the seed germination process in the next generation (Begcy et al., 2018). BMVE treatment of developing seeds leads to higher root and shoot growth in the next generation regardless of temperature treatment, suggesting that BMVE triggers biological processes that lead to increased seed vigor.

## Discussion

Because treatment of dry seeds reportedly increases final yield of several crop plants, a reasonable hypothesis is that BMVE affects early stages of seedling development, with positive effects extending throughout plant growth (Yokoyama et al., 2015; Kamtec LLC, unpublished). To address the earlier issues of inconsistencies in our own evaluation of BMVE seed treatment (Singh, 2018), we developed a new treatment protocol based on the triphasic model of seed germination (Bewley, 1997; Yang et al., 2007). Phase 1 corresponds to the rapid uptake of water by a dry seed and lasts up to 20 hours after imbibition (HAI) (Bewley, 1997; Howell et al., 2009). Phase II lasts from 20 - 50 HAI and is considered the most critical, as most of the metabolic reactions required for germination occur during this window (Yang et al., 2007; He and Yang, 2013). Phase III begins 50 HAI onwards in which another rapid water uptake takes place followed by radical protrusion (Yang et al., 2007). We hypothesized that as a beneficial seed treatment BMVE targets phase II. To enhance treatment efficacy, we imbibed the seeds for 24 hours prior to BMVE treatment. In addition to targeting the early metabolically active phase II, this allowed for the selection of seeds at the same stage of germination for subsequent BMVE treatment, minimizing the confounding effects of natural variability in germination rate among seeds. We also optimized the BMVE concentration for treatment under this condition. Together, this gave repeatable beneficial effects of BMVE on wheat and rice seedling shoot and root length as early as 48 HAT (24 HAT for wheat shoots) in our relatively small-scale laboratory experiments.

When BMVE treated wheat seedlings that were grown in well-watered soil for up to 52 days after transplanting, the advantage observed during the early seedling growth was not evident for the parameters measured during the vegetative growth phase. However, we did not obtain final grain yield data, which might still have shown a significant difference in the end. In contrast to plants with sufficient water, the benefit of BMVE treatment became evident as early as 35 days after transplanting (number of leaves and tillers) when the plants began to experience drought conditions (**Figure 3**). Total biomass determined by RGB imaging corroborated this result, beginning at 49 DAP, when drought had become severe. The higher biomass observed for BMVE treatment under the WL condition could be primarily due to increased tillering, although the final tiller number for BMVE treatments under WL conditions were still lower than that of the WW treatment. Further testing that includes final yield data and grain parameters would help to better understand the benefits of BMVE seedling treatment. We also acknowledge that other crop species may not respond similarly to BMVE.

These results suggest that BMVE is most beneficial under abiotic stress conditions, based both on our physiological experiments and from the number of stress-related genes affected by BMVE. DCPTA is another STA that has been reported to ameliorate drought stress following foliar application (Xie et al., 2017). Anecdotal observations also suggest that these compounds are more likely to be beneficial under stress conditions in field experiments (Kamtec, LLC, unpublished). Differential environmental stress conditions may partly explain the discrepancies in past field studies that sometimes showed no beneficial effect of STA treatment.

Our demonstration of altered seedling growth parameters coupled with the transcriptome results suggest that early changes in gene expression mediate the BMVE effect. Specifically, transcripts associated with photosynthesis, carbohydrate metabolism, cell growth and hormones regulating growth were all increased 24 h after BMVE treatment relative to the control. Several peroxidase and cytochrome protective enzyme transcripts were also elevated. Early seed germination leads to a nutrient deprived state, resulting in rapid mobilization and oxidation of nutrient reserves. ROS species are a detrimental by-product of these oxidation processes (Thakur and Vasudevan, 2019). To facilitate seed germination, antioxidant systems are activated to scavenge free radicles (Steinbrecher and Leubner-Metzger, 2017; Marks et al., 2022). Hence, antioxidant activity is considered a reliable indicator of robust seed germination and seedling performance (Corbineau, 2012). One of the observed results of seed priming is higher activity of antioxidant defense mechanisms, which presumably contributes to more rapid and synchronous germination and more uniform crop stands (Jisha et al., 2013; Hussain et al., 2016). Notably, our transcriptome analysis results revealed that BMVE treatment not only increased the expression of genes that are involved in antioxidant and detoxification activities but also reduced the expression of those that promote the production of free radicles, in particular, H_2_O_2_ (**Figures 6**, **7**). This suggests that BMVE treatment might mimic some of the priming effects seen during early seedling growth, which ultimately enables accelerated development of seedlings. It would be important in future studies to evaluate whether any of the transcriptomic changes we observed persist into the mature plant, or if other genes change their pattern of expression as plants mature.

We cannot rule out the possibility that BMVE also has a direct biochemical effect on early seedling metabolism that does not require altered gene expression. Yokoyama and Keithly (1991) proposed that DCPTA [2-(3,4-dichlorophenoxy) triethylamine] modifies carotenoid content in plants and mold (*Phycomyces blakesleeana*) directly by modifying enzyme activity, as well as indirectly by changing gene expression for an extended period. Evidence for the latter is that carotenoid changes persisted after several serial transfers to culture media lacking DCPTA. Gao et al. (2022) reported that foliar spraying of mung bean plants resulted in increased SOD, POD, and CAT enzyme activities within 7 days, although the mechanism for this increase was not reported.

One of the more notable trends seen from the transcriptome analysis was how BMVE treatment changes the normal temporal trajectory of gene expression (**Figure 7**). For instance, expression of cytochrome P450s (*CYP86A7-2*, *CYP96E1*, *CYP76M1*) and PRX (*LOC_Os04g51300*) declines over 24 h for untreated seedlings (C0 vs. C24), while BMVE maintains these transcripts at 24 h. Similar trends were observed for other genes that are involved in ROS scavenging (*GABA-T*), development (*PSK5*), carbohydrate metabolism (*GLHL17* and *DIP3/XIP2*), and hormonal activities (*SAUR55* and *GID1L3*). In contrast, BMVE treatment greatly reduces the expression of some genes that exhibit minimal reduction over time for the control seeds. For instance, expression of the H_2_O_2_ promoting genes (*CDP3*, *OXO3*, and *OXO4*) declines normally as evident from the control seeds C0 vs. C24) but BMVE treatment led to a further reduction in transcript levels (**Figure 7**). A similar pattern was observed for the upregulated genes in all other classes where the expression normally increases over time, but even higher levels were usually observed for BMVE treated seedlings. Higher activity of antioxidants enhance plant growth by fostering their tolerance capabilities, suggesting this is an important mechanism of BMVE activity (Gill and Tuteja, 2010).

Most transcripts for stress associated genes (response to stress, abiotic/water/chemical) were reduced for BMVE treated seedlings (**Figures 6**, **7**). For example, *RERJ1*, *bHLH148*, *NAC22*, *LEA3*, *LEA1*, and *ERF104* transcripts increased over time for control seedlings, however, the levels for B24 were lower than for C24 (**Figure 7**). For other stress associated genes, such as *Soul haem-binding*, *HSP101*, *LEAs*, *RAB21*, *ABCG6*, and *NCED4*, transcript level declined over time for control seeds, but the reduction was even greater for BMVE treated seedlings (**Figure 7**). There were exceptions to this trend. Genes associated with drought response (*MYB60*, and *LOC_Os11g03440*, another MYB60 ortholog), and abiotic stress (LOC_Os02g14680, *ABCG11*, *LOC_Os05g06140*) and biotic stress tolerance (*isoflavone reductase*, *PS1*/*OSC7*) exhibited higher transcript levels for B24 compared to C24 (**Figures 6**, **7**). However, it should be noted that these genes could also have roles that are beneficial to growth in the absence to stress, in which case their upregulation would be consistent with the physiological data.

It might seem counterintuitive that reduction in several stress associated genes is correlated with enhanced growth. However, this may not be causal to the BMVE effect, rather, a result of BMVE treated seedlings experiencing less stress due to the enhanced antioxidant activity. Although antioxidant levels were not directly measured in this study, the BMVE mediated upregulation of genes associated with antioxidant mechanisms could explain the reduced expression of stress associated genes.

We cannot distinguish between the possibility that the BMVE-potentiated changes in transcript abundance are a direct cause of enhanced seedling growth, or whether the changes simply reflect a more advanced stage of growth at B24 versus C24. This may well be the case for several gene transcripts associated with development, chloroplast, and carbohydrate metabolism that normally increase (C0 vs. C24), but their levels are even higher for B24 (faster growing). Gao et al. (2022) observed that increased photosynthetic capacity in mung bean following foliar application of DCPTA was correlated with increased transcript levels for sucrose synthase, fructokinase, and beta-fructofuranosidase. However, whether DCPTA directly caused the increased gene expression in this case is unclear due to the 7 d delay between DCPTA application and transcript analysis.

We suspect that early changes in some genes we observed are causal for enhanced seedling growth. As mentioned earlier, ABA negatively regulates seed germination and the ABA synthesis enzyme transcript *NCED4* normally declines during germination (**Figure 8**) (Huo et al., 2013). Our results showed that BMVE treatment leads to an earlier repression of *NCED4* transcripts (nearly 3-fold within 2 HAT) compared to no loss for the control at 2 HAT. (2 HAT corresponds to 24 h imbibition, plus 3 h BMVE treatment, followed by 2 h further growth). By 6 HAT the control transcript level had declined to a similar level as the BMVE treatment. It seems unlikely that accelerated seedling growth was itself responsible for the BMVE mediated decline in *NCED4* transcript at 2 HAT, since rice shoot and root growth enhancement were only detectible at 48 HAT. This suggests that early hormonal changes in response to BMVE may regulate metabolic responses that then translate to faster growth. Genes associated with increased antioxidant activity following BMVE treatment may also have a causative role, as similar activity is associated with seed priming treatments that enhance germination and seedling vigor. Future studies could explore additional genes at early time points to help identify candidate causal regulatory genes in the BMVE response.

Robust seed germination and seedling growth are dependent on events that occur early in seed development on the mother plant (Lopes and Larkins, 1993; Luo and Tucker, 2001). Proper endosperm development is crucial as it provides mechanical support during early embryo growth and it is a storage site for nutrient reserves that facilitate subsequent seed germination (Lopes and Larkins, 1993). Exposure to heat stress during endosperm development alters the initiation of endosperm cellularization, impacting mature grain characteristics (Folsom et al., 2014; Chen et al., 2016; Begcy et al., 2018).

The positive association of BMVE seedling treatment with stress responses prompted us to examine its effect on seed development under heat stress. Developing seed weight showed no change between the control and BMVE treatment when the duration of HS imposed was up to 48 hours (**Figure 9B**). The probable reason behind the higher seed weight at 48 HS as compared to the ambient conditions could be the precocious endosperm cellularization caused by moderate heat stress (Chen et al., 2016; Begcy et al., 2018). Under the moderate heat stress used in our study cellularization of seeds begins around 48 HAF and completes by 72 HAF, respectively, whereas under ambient conditions it completes by 96 HAF (Folsom et al., 2014; Chen et al., 2016). For 96 HS, BMVE application significantly improved the developing grain weight. This suggests that BMVE might be accelerating seed development under HS conditions. Interestingly, the mature seed weight, which was not changed for 24 and 48 HS between BMVE and control treatments, showed a significant drop for the control seeds under 96 HS. This is in line with a previous study that reported precocious cellularization under HS results in smaller seed size and reduced mature grain weight (Folsom et al., 2014). In contrast, BMVE treatment restored the mature grain weight to the ambient levels for 96 HS seeds (**Figure 9**). This suggests that during the comparatively longer 96 HS exposure only the control treated seeds would have gone through the precocious cellularization, and the BMVE treated seeds might have developed normally. Future evaluation of developing and mature seeds under scanning electron microscope should validate the processes related to cellularization, grain filling, and starch composition.

BMVE treatment during seed development increased grain length under both temperature treatments (**Table 1**). Grain width and area exhibited no change between control and BMVE treatment but were significantly lower for the control seeds developed under HS. Mohammed et al. (2013) showed that exposure to higher temperatures during the rice reproductive phase reduces the thickness of mature grains. The decline we observed for mature weight for the control seeds that developed under HS could be due to reduced grain size parameters including width and area (**Figure 9B**, **Table 1**). This showed that the BMVE treatment improves grain length in general and negates the detrimental impact of HS on the grain width and area.

Seeds that developed under 96 HS exhibited early germination (**Figure 10A**), probably due to altered ABA sensitivity or increased starch levels caused by exposure to non-terminal moderate HS (Begcy et al., 2018). However, despite the temperature conditions that the seeds were exposed to during the post-fertilization period, BMVE treated seeds exhibited faster germination rates as compared to control treated seeds (**Figure 10**). This led to significantly higher root and shoot lengths in both temperature treatment groups. For the control seedlings, the significantly higher shoot length under HS further supported the growth promoting effect of moderate HS explained by Begcy et al. (2018). Overall, the evidence suggests that irrespective of the temperature treatment, BMVE applied soon after fertilization alters metabolic activity in a way that enhances seed germination vigor in the next generation. Exploring gene expression changes in developing seeds could elucidate the mechanisms for this activity.

One caveat in our results is that, as whole panicles were sprayed with BMVE we do not know whether BMVE had a direct effect on the developing seeds. Panicles are a significant source of photosynthate, both transported and locally produced, so it is possible that the treatment affected the plant’s capacity to supply resources to developing seeds. This would be in line with reports that STAs do enhance photosynthetic processes when applied to mature plants (Keithly et al., 1990a, b; Yokoyama et al., 2015; Gao et al., 2022.).

In summary, the optimized experimental conditions we developed provide convincing molecular and biological evidence that BMVE does indeed have efficacy in plant growth enhancement. Because of its laborious nature, the protocol is not suitable for large scale industrial applications, but it identifies conditions under which further exploration of the mechanism of STA activity can be explored. Increased expression of genes involved in antioxidant activity and early modification of genes associated with hormonal regulation may be key players driving accelerated seedling growth. BMVE application during early seed development also enhances germination and seedling growth in the next generation, under both ambient and HS conditions. To our knowledge this is the most comprehensive study tying gene regulatory effects to plant growth enhancement by STAs. It provides a base of new knowledge that points to avenues to further explore the molecular mechanisms of STA activity, which could lead to the development of practical applications.

## Data availability statement

The original contributions presented in the study are included in the article/**Supplementary Material**, further inquiries can be directed to the corresponding author/s.

## Author contributions

PS: Conceptualization, Formal analysis, Funding acquisition, Investigation, Methodology, Project administration, Supervision, Writing – review & editing. JSD: Data curation, Investigation, Methodology, Writing – original draft. YS: Formal analysis, Writing – review & editing. CZ: Formal analysis, Supervision, Writing – review & editing. CP: Funding acquisition, Supervision, Writing – review & editing. HW: Conceptualization, Investigation, Methodology, Supervision, Validation, Writing – review & editing.
