# Supplementary figures and images for: Growth and transcriptional response of wheat and rice to the tertiary amine BMVE
Source: Front Plant Sci. 2024 Jan 10;14:1273620. doi: 10.3389/fpls.2023.1273620 (PMC10806070; doi:10.3389/fpls.2023.1273620)

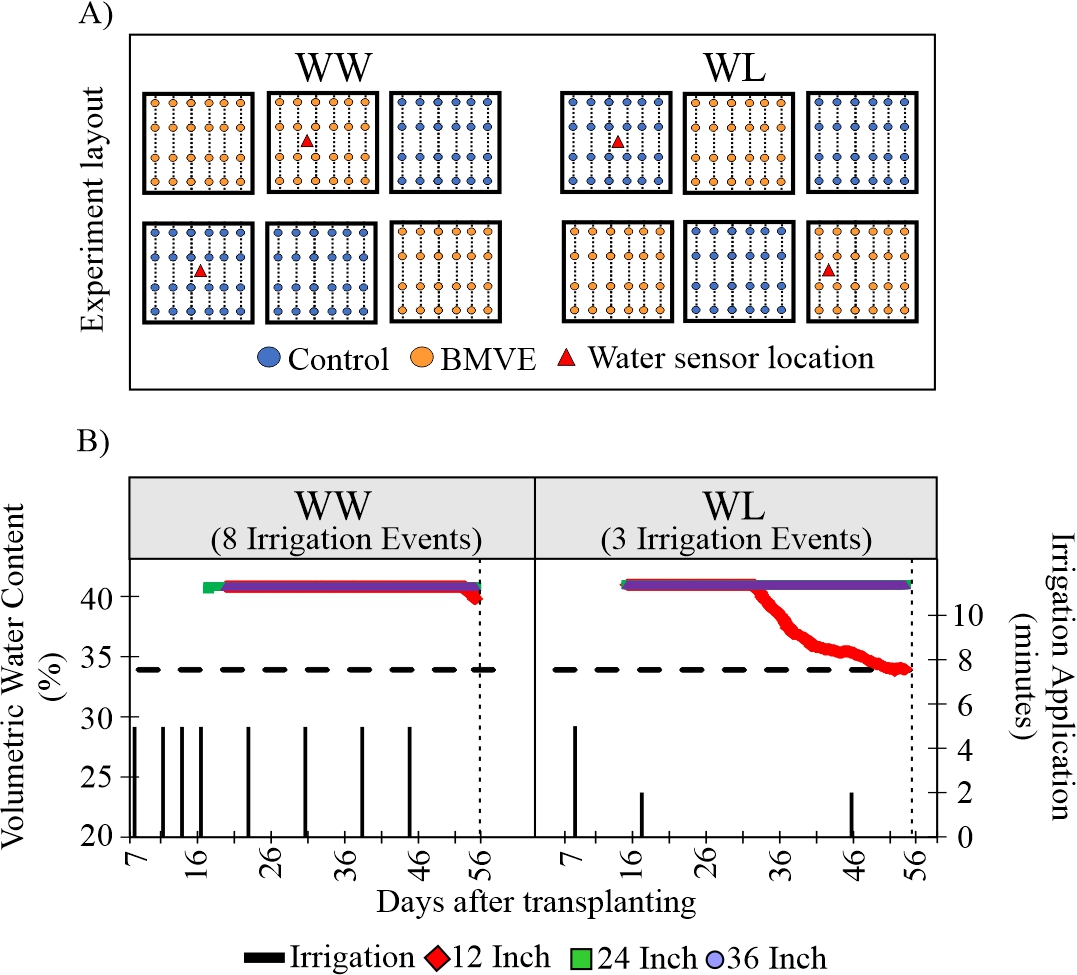

Supplement: Supplementary file 1 [file Image_1.jpeg]
