# Supplementary material for: Growth and transcriptional response of wheat and rice to the tertiary amine BMVE
Source: Front Plant Sci. 2024 Jan 10;14:1273620. doi: 10.3389/fpls.2023.1273620 (PMC10806070; doi:10.3389/fpls.2023.1273620)
Supplement: Supplementary file 2 [file Table_1.docx]

**Supp Table 1. Unique gene IDs recovered from the GO enrichment analysis**

| Class | MSU_Ids | Gene_Symbol or Family | Expresion B24 vs C24 |
| --- | --- | --- | --- |
| Peroxidase and Cytochrome P450/Detoxification | LOC_Os04g48200 | *DIM/DWF1* | Up |
|  | LOC_Os01g63540 | *CYP86A7-2* | Up |
|  | LOC_Os01g08810 | *CYP96E1* | Up |
|  | LOC_Os08g36310 | *CYP76M1* | Up |
|  | LOC_Os10g02040 | *PRX125* | Up |
|  | LOC_Os05g06970 | *PRX72* | Up |
|  | LOC_Os04g51300 | *PRX. Unknown* | Up |
| Chloroplast associated | LOC_Os03g47610 | *THIC* | Up |
|  | LOC_Os04g16680 | *SBPase* | Up |
|  | LOC_Os01g73540 | *YGGT family protein* | Up |
|  | LOC_Os03g39610 | *Lhcb2* | Up |
|  | LOC_Os07g11110 | *RNA binding* | Up |
| Extracellular/cell wall/Development | LOC_Os03g58980 | *GLP3-7* | Up |
|  | LOC_Os07g03710 | *PR1A* | Up |
|  | LOC_Os11g42500 | *Dirigent* | Up |
|  | LOC_Os03g47230 | *PSK5* | Up |
|  | LOC_Os06g05550 | *GELP74* | Up |
| Hormone associated | LOC_Os06g04590 | *SAUR 24* | Up |
|  | LOC_Os02g24700 | *SAUR 8* | Up |
|  | LOC_Os09g37460 | *SAUR 51* | Up |
|  | LOC_Os09g37500 | *SAUR 55* | Up |
|  | LOC_Os05g15630 | *BLE3* | Up |
| Response to stress, abiotic/biotic/water/chemical | LOC_Os01g63210 | *Soul haem-binding* | Down |
|  | LOC_Os05g44340 | *HSP101* | Down |
|  | LOC_Os04g23550 | *RERJ1* | Down |
|  | LOC_Os03g53020 | *BHLH148* | Down |
|  | LOC_Os03g04070 | *NAC22* | Down |
|  | LOC_Os05g46480 | *LEA3/EM1* | Down |
|  | LOC_Os11g26750 | *LEA26* | Down |
|  | LOC_Os11g26780 | *LEA28* | Down |
|  | LOC_Os11g26760 | *LEA27* | Down |
|  | LOC_Os05g28210 | *LEA21* | Down |
